# The Spinal Curvature of Three Different Sitting Positions Analysed in an Open MRI Scanner

**DOI:** 10.1100/2012/184016

**Published:** 2012-11-25

**Authors:** Daniel Baumgartner, Roland Zemp, Renate List, Mirjam Stoop, Jaroslav Naxera, Jean Pierre Elsig, Silvio Lorenzetti

**Affiliations:** ^1^Institute for Biomechanics, ETH Zurich, Zurich, Switzerland; ^2^School of Engineering, Winterthur, Switzerland; ^3^Röntgeninstitut Zürich-Altstetten, Zurich, Switzerland; ^4^Spine Surgery, 8700 Küsnacht, Switzerland

## Abstract

Sitting is the most frequently performed posture of everyday life. Biomechanical interactions with office chairs have therefore a long-term effect on our musculoskeletal system and ultimately on our health and wellbeing. This paper highlights the kinematic effect of office chairs on the spinal column and its single segments. Novel chair concepts with multiple degrees of freedom provide enhanced spinal mobility. The angular changes of the spinal column in the sagittal plane in three different sitting positions (forward inclined, reclined, and upright) for six healthy subjects (aged 23 to 45 years) were determined using an open magnetic resonance imaging (MRI) scanner. An MRI-compatible and commercially available office chair was adapted for use in the scanner. The midpoint coordinates of the vertebral bodies, the wedge angles of the intervertebral discs, and the lumbar lordotic angle were analysed. The mean lordotic angles were 16.0 ± 8.5° (mean ± standard deviation) in a forward inclined position, 24.7 ± 8.3° in an upright position, and 28.7 ± 8.1° in a reclined position. All segments from T10-T11 to L5-S1 were involved in movement during positional changes, whereas the range of motion in the lower lumbar segments was increased in comparison to the upper segments.

## 1. Introduction

During daily life, increasing amounts of time are spent in a sedentary position. In industrial countries, more than 75% of all office workers sit for periods of more than seven hours [1]. In contrast to walking and running, muscles are not actively used during sitting. The muscular function is replaced by the supporting effect of the seat. Muscular inactivation over a long period of time leads to a weakening of the corresponding muscles. Approximately half of all office workers are affected by back problems [2], and recent trends show an increase in this number. Current research is therefore focussed on sitting in relation to discomfort and pain [1]. Grimmer et al. [3] found that adolescents have high rates of back pain which are medically verifiable and follow into adulthood. Accelerated degeneration of the spine due to long-term sitting results in a higher number of disc protrusions in the elderly [4]. Further negative effects such as muscle clenching, nerve irritation, reduced blood circulation due to compressed veins, or narrowing of the respiratory organs may appear [4–7]. Such diseases may potentially cause chronic health problems in the elderly. A study by Katzmarzyk et al. [8] showed a higher incidence of cardiovascular disease and a higher risk of mortality for office workers compared with physically active working people, independent of their physical activity level during leisure time. These data were confirmed in a similar study performed by Patel et al. [9]. A long-term sitting position therefore seems to be one of the highest risk factors for developing future health problems. This fact is also supported by a recent study of Dunstan et al. [10]. They established that short bouts of walking during sitting time lower postprandial glucose and insulin levels in overweight/obese adults. Dunstan et al. [10] finally concluded that this may improve glucose metabolism and potentially be an important public health and clinical intervention strategy for reducing cardiovascular risk.

Recent developments in the field of ergonomic office furniture allow different types of movements, such as forward and backward inclination as well as lateral tilting of the seat [11]. A less constrained seat system leads to an alternating load on the spine, particularly on the intervertebral discs. Active but controlled sitting is believed to activate muscles and supporting structures and therefore prevent static loads acting on joints, ligaments, and tendons. It has been shown that an alternating sitting position significantly enhances muscular activity [12].

Continuous upright sitting has been shown to be undesirable since the 1960s. Novel solutions with adjustable backrests or seats that alternate kyphosis and lordosis angles have been presented. The kneeling chair represented one of the first sitting concepts that significantly influenced spinal posture. For example, Bennett et al. [13] found an increased lumbar curvature when sitting in a Balans Multi-Chair (kneeling chair) compared to sitting upright in a straight-backed chair. Other concepts included applying very small active rotational seat movements using motor-driven actuation, which resulted in a twisting of the spine along the vertical axis within the natural range of movement of individual intervertebral discs [14]. These dynamic stimuli apparently influenced the length of the spine after sitting for a certain period of time. This continuous passive motion concept was previously published by Reinecke et al. [15] and was thoroughly investigated in later studies by Lengsfeld et al. [16] and van Deursen et al. [17]. Recently, this actively steered chair was the focus of a biomechanical investigation by van Dieën et al. [18].

The lordosis angle of different body postures and its effect on lumbar biomechanics have often been the focus of spinal research. Bridger et al. [19] concluded that the lordosis angle was smaller in a sitting position compared to a standing position. A forward tilted sitting position was therefore suggested in order to achieve similar lordosis angles as in standing. A reclined position reduces the load on the intervertebral discs and on the back muscles by an increased lordosis angle, which was shown by Colombini et al. [20]. Graf et al. [21] demonstrated a certain discomfort with a tilting seat angle of more than 15°. Apparently, the biomechanical analysis of the lordosis angle is of relevance when determining the influence on posture and wellbeing. A more accurate analysis of the behaviour of single functional spinal units instead of the lordosis angle could be of advantage. 

Seat systems that allow several sitting positions have a significant influence on posture. The assessment of posture is hindered by the fact that the spine is positioned below soft tissue and the skin surface. The location of a single vertebra can only be assumed by the external shape of the thorax or by palpating the spinous processes on the skin surface. Hence, a reproducible analysis method to quantify the location of single vertebrae is needed. Magnetic resonance imaging (MRI) techniques are therefore valuable for displaying the exact vertebral position in sedentary positions [22, 23]. By use of an upright, open MRI scanner, acquisition of 3-D data in the standing or sitting position is possible. In this way, the spine of wheelchair users has been investigated in a study by Linder-Ganz et al. [23]. Savage et al. [24] and Videman et al. [4] correlated the clinical diagnosis displayed by MR images with the occurrence of symptomatic low back pain. Bertschinger et al. [22] compared sedentary patients in an open MRI scanner versus a traditional MRI scanner, in which patients have to lie down. In contrast to the standardised lying position in the closed-magnet unit, the spinal column is loaded by the gravitational weight of the thorax in the upright position. Thus, a sedentary position seems to be more clinically relevant in performing an accurate clinical diagnosis [25]. Consequently, the analysis of variable sedentary positions on office chairs and the influence of these positions on spinal biomechanics can be accurately analysed with an open, upright MRI scanner.

Dynamic or active sitting occurs when a chair enables the seated user to move in different planes. Flexibility and movement during sitting may be beneficial to wellbeing and allow different movement tasks to be performed. In our specific case, dynamic movement denotes a forward tilting mechanism of the seat pan. In particular, a higher degree of freedom for the hip flexural angle is provided which substantially influences the lumbopelvic mechanism and consequently the whole thoracolumbar region of the spine. 

The aim of this study is to analyse the spinal shape and, in particular, the position of single intervertebral bodies in relation to the sitting posture. The results may help to evaluate novel designs of backrests from a physiological point of view.

## 2. Material and Methods

### 2.1. Subjects

Six subjects (three females and three males, average age: 32 years (range 23 years to 45 years), average height: 1.74 m (range 1.64 m to 1.78 m), average weight: 68 kg (range 60 kg to 77 kg)) were measured in the three different positions. The subjects needed to have a maximum trunk width of 48 cm (distance from left to right shoulder) to have enough space in the MRI scanner. Clinical and therapeutic interventions relating to back problems were exclusion criteria. These criteria included previous back surgeries, diagnosed postural deformities in the sagittal or frontal plane, and presence of ferromagnetic implants in the body. Before measurements were made, metallic objects such as necklaces or watches were removed.

No financial compensation was provided for participation in the study. A survey was filled out by every subject describing body parameters and history of back problems and clinical interventions. The study was approved by the ethics commission of the ETH Zurich (no. EK 2010-N-27).

### 2.2. Investigated Positions

**Figure 1:**
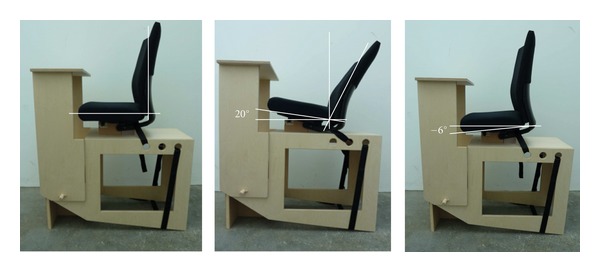
MRI-compatible chair in the three positions: upright (left), reclined (middle), and forward inclined (right).

The spinal posture and the position of the lumbar and lower thoracic vertebrae were analysed. Subjects were positioned on a specifically designed, MRI-compatible chair in the upright MRI scanner. The chair did not contain any ferromagnetic assemblies to exclude image artefacts. The duration of the scanning period depended on the size of the subject and was approximately three to five minutes per position. During measuring, the subject had to maintain a static position as far as possible. Three different chair positions were analysed (Figure 1).

**Upright (up)**

The lumbar spine was in contact with the backrest, but no force was transmitted. The hands were placed on the legs.

**Reclined (re)**

The back had contact with the whole backrest of the chair, the hands were placed on the legs, and the head was kept looking straight ahead. The subject was able to choose the most individually appropriate position.

**Forward Inclined (fi)**

The back had no contact with the backrest, and the upper body was supported by the arms lying on a table in front of the subject.

A randomised sequence of the positions for every patient was performed. While changing from one position to another, a recovery time of five to ten minutes was given. During that time, the subjects were requested to walk around and relax the musculoskeletal system.

### 2.3. Data Acquisition and Measuring Sequence

Measurements were taken in the Upright MRI Center, Zurich, with the FONAR Upright MRI scanner (0.6 Tesla). T2-weighted sagittal images were taken with a repetition time of 3435 ms, an echo time of 110 ms, and a layer thickness of 4 mm. The resolution was 240 × 240 pixels in an image plane of 380 × 380 mm.

**Figure 2:**
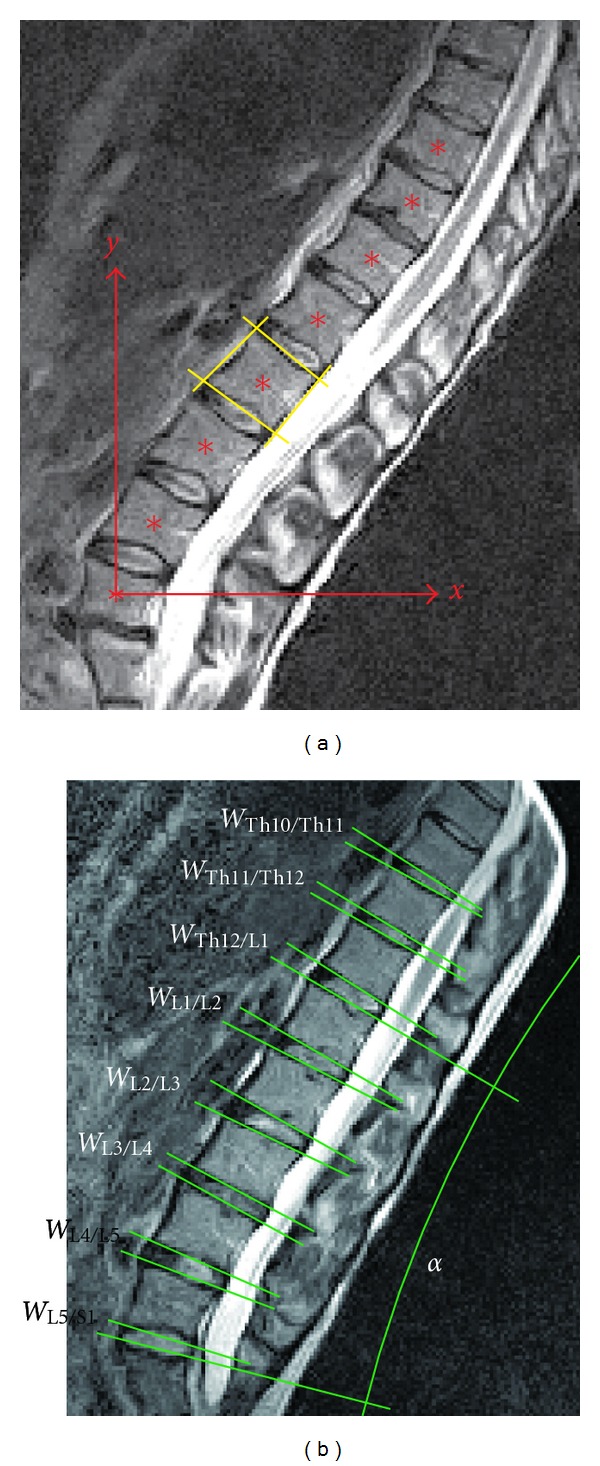
(a) The coordinate system (red arrows) and the quadrangle built by the two endplates and the ventral and dorsal margins of the vertebral body (yellow straight lines) to determine the coordinates of the vertebral midpoints (red stars). (b) The wedge angles of the intervertebral discs (*W*_L5/S1_, *W*_L4/L5_,…) and the lordotic angle *α*.

In total, 15 sagittal sections were obtained for a vertebra with a 60** **mm width. The scans were captured along the vertical axis of the spine in sagittal sections. In a lateral view, the cross-section of the single discs and their adjacent vertebrae are displayed. Discs and vertebrae can be easily separated due to the different contrasts, which is a result of the increased water content of the discs compared to the vertebral bone. The vertebral end plates between the discs and the vertebrae are displayed in a dark colour (Figure 2(a)). 

### 2.4. Data Analysis

The following parameters were evaluated based on the MR images of the most central section (median plane of the body).

**Coordinates of the Midpoints of the Vertebrae**

A coordinate system was placed corresponding to the main direction of the MR image with the origin through the lowest vertebra L5 (Figure 2(a)). The *x*- and *y*-coordinates were determined for all three sitting positions in the sagittal view for each vertebra (from L5 to Th10). The coordinates were determined based on the centre of a quadrangle built by the two endplates and the ventral and dorsal margins of the vertebral bodies.

**Wedge Angles of the Intervertebral Discs**

The wedge angles (from L5/S1 to Th10/Th11) were determined according to an established, clinical evaluation [26]. A tangent line was placed on the ventral and dorsal edges of each vertebra. The wedge angle was defined in between two lines of adjacent vertebral bodies (Figure 2(b)).

**Lordotic Angle**

The angle between the tangent line on the upper L1 endplate and the tangent line on the upper sacrum S1 endplate is defined as the lordotic angle *α* (Figure 2(b)).

**Convention**

A lordosis was defined as a positive angle and a kyphosis as a negative angle.

MegaCAD 2D software (Version 2011, MegaCAD-Center GmbH, Oberweningen, Switzerland) was used to examine the coordinates and angles.

### 2.5. Statistical Analysis

All statistics were determined using IBM SPSS Statistics (Version 19, SPSS Inc., Chicago, IL, USA). The statistical significance level was set at *P* < 0.05.

The averages of the wedge angles and the lordotic angle of the three positions (up, re, fi) were compared with the Wilcoxon tests in a crosswise manner.

## 3. Results

### 3.1. Coordinates of the Midpoints of the Vertebrae

**Figure 3:**
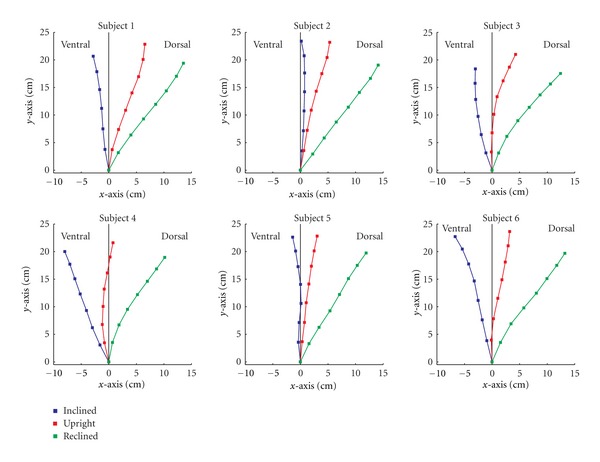
Coordinates of the midpoints for the vertebrae of the three positions. All curves are related to the same origin, represented by the midpoint of L5.

Changes in the position and shape of the spine occurred during the three different sitting positions (Figure 3). The reclined sitting posture resulted in similar positions of the vertebrae for all subjects. In contrast, the largest differences in the position of the midpoints between the subjects occurred when patients were in the forward inclined sitting posture. In the upright sitting posture, five subjects had a slightly dorsally located spine. Only one subject (Subject 3) showed a ventral configuration of the vertebrae, especially in the lumbar region. For all subjects, the shape of the lumbar spine was similar during the upright and the inclined positions.

A line through the midpoints of the vertebrae, approximating the direction of the spine in the reclined position, was dorsally ascending with an angle between 30° and 40° relative to the vertical axis. The upright position was dorsally ascending with a large variability for all subjects.

### 3.2. Wedge Angles

**Figure 4:**
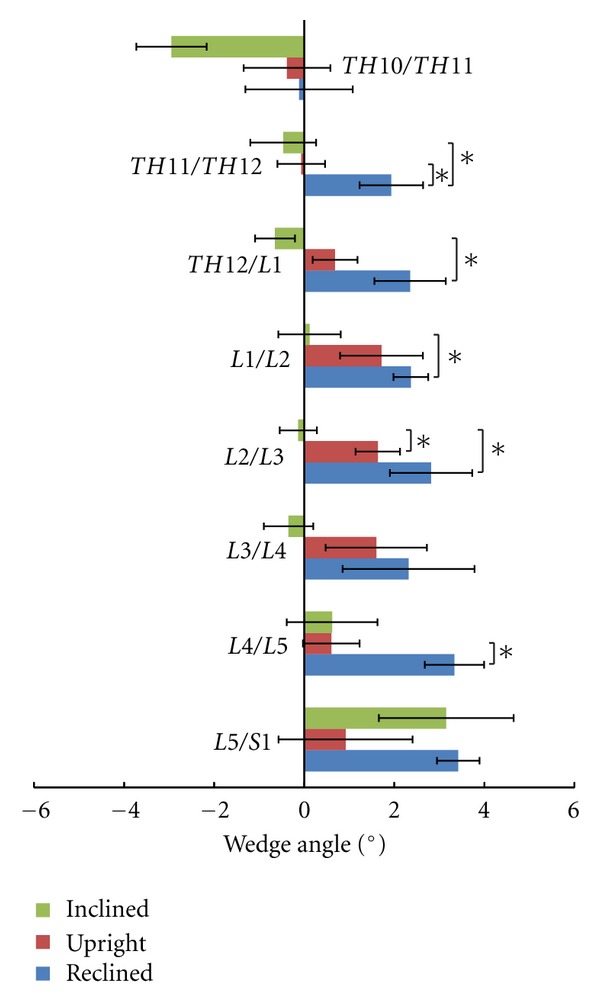
Mean wedge angles and their standard error of the intervertebral discs. *Significant differences between positions.

The maximal measured mean wedge angle was 3.4 ± 1.2° (mean ± standard deviation) for the lowest lumbar segment in the reclined position. The maximal mean changes within one segment were 3 ± 2° (mean ± standard deviation) from the forward to the reclined position for the wedge angle TH12/L1. Generally, a change in position was visible for all segmental heights, and some were significant (Figure 4). A general trend of a uniform movement pattern was not observed for the six subjects. Two subjects reached their maximum wedge angle in the upright position, while all others reached this angle in the reclined position.

### 3.3. Lordotic Angle

**Figure 5:**
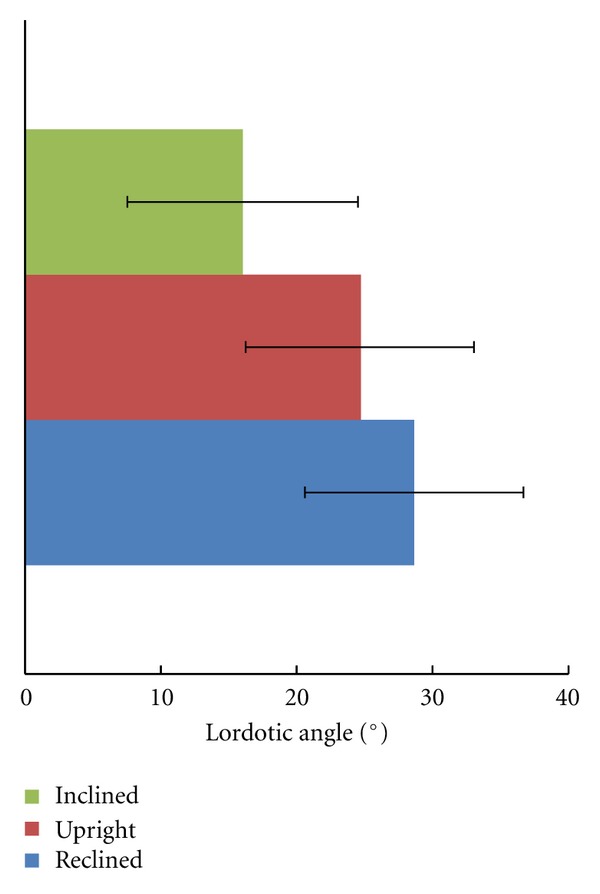
Mean lordotic angles (*α*) and their standard deviation for the three positions.

The mean lordotic angle *α* for the forward inclination was 16.0 ± 8.5° (mean ± standard deviation), for the upright position was 24.7 ± 8.3°, and for the reclined position was 28.7 ± 8.1° (Figure 5). High individual differences were observed for the lordotic angle. These large interindividual differences were also observed for the reclined position, although a standardised backrest was used. The lordotic angles were not significantly different between the three positions.

## 4. Discussion 

All lumbar and lower thoracic intervertebral discs are involved in positional changes and contribute to the change in the spinal shape. These findings were revealed by applying the clinical evaluation method to determine the wedge angles. No specific segment can be identified in which the majority of the movement is performed. Slight trends in the absolute wedge angles of the intervertebral discs could be determined. The angles in the forward inclined and reclined positions seem to be higher for the lower lumbar vertebral discs and decrease towards the tenth thoracic vertebra. In contrast, individual differences between the subjects were much higher for the upright position compared with the other positions. No general movement pattern caused by changing positions was detected. High individual differences are visible, although the geometry of the test chair was standardised for all tested subjects. 

The current study was performed with only 6 subjects, which represents the main limitation. Only some of the analysed spinal angles were significantly different. To provide more statistically significant data, more subjects would be required. However, some general statements about the behaviour of the vertebral discs of the lower back could be made.

In conclusion, the wedge angles and the position of the vertebral bodies change between the three described sitting positions. As a result, the load condition of the intervertebral discs changes. This is assumed to stimulate the metabolism of the intervertebral discs[27]. Even slight changes in the position cause a change in the disc loading. Positional changes from an upright to a reclined or forward inclined sitting position may therefore have a positive effect on the biological nutrition processes of the spine.
